# Increased Male-Male Mounting Behaviour in Desert Locusts during Infection with an Entomopathogenic Fungus

**DOI:** 10.1038/s41598-017-05800-4

**Published:** 2017-07-18

**Authors:** Lisa M. Clancy, Amy L. Cooper, Gareth W. Griffith, Roger D. Santer

**Affiliations:** Institute of Biological, Environmental, and Rural Sciences, Aberystwyth University, Penglais Campus, Aberystwyth, Ceredigion SY23 3FG UK

## Abstract

Same-sex sexual behaviour occurs across diverse animal taxa, but adaptive explanations can be difficult to determine. Here we investigate male-male mounting (MMM) behaviour in female-deprived desert locust males infected with the entomopathogenic fungus *Metarhizium acridum*. Over a four-week period, infected locusts performed more MMM behaviours than healthy controls. Among infected locusts, the probability of MMM, and the duration of time spent MMM, significantly increased with the mounting locust’s proximity to death. In experimental trials, infected locusts were also significantly more likely than controls to attempt to mount healthy males. Therefore, we demonstrate that MMM is more frequent among infected than healthy male locusts, and propose that this may be explained by terminal reproductive effort and a lowered mate acceptance threshold in infected males. However, during experimental trials mounting attempts were more likely to be successful if the mounted locusts were experimentally manipulated to have a reduced capacity to escape. Thus, reduced escape capability resulting from infection may also contribute to the higher frequency of MMM among infected male locusts. Our data demonstrate that pathogen infection can affect same-sex sexual behaviour, and suggest that the impact of such behaviours on host and pathogen fitness will be a novel focus for future research.

## Introduction

Same-sex sexual behaviours comprise ‘interactions between same-sex individuals that also occur between opposite-sex individuals in the context of reproduction’, and are distinct from sexual preference and orientation^1^. Such behaviours are widespread across diverse animal taxa, and a range of adaptive and non-adaptive explanations for them have been proposed^1, 2^. Whilst opposite-sex sexual behaviours are known to be modulated by pathogen infection in ways that are adaptive for the pathogen or the infected individual, the effects of pathogen infection on same-sex sexual behaviours, and explanations for it in this context, have so far not been explored.

Pathogen infection can have a variety of effects on opposite-sex sexual behaviour. For example, immune-challenged male mealworm beetles, *Tenebrio molitor*, increase their investment in pheromone signals that attract females^3^, and female *Gryllus lineaticeps* crickets infected with the parasitoid fly *Ormia ochracea* show reduced selectivity in mate choice^4^. For infected individuals, such behavioural modulations may be adaptive because increased reproductive effort is expected when there is a low probability of being able to reproduce successfully in the future (‘terminal investment’)^3, 5–8^, and a lower threshold for mate acceptance is expected when the costs of incorrectly rejecting a suitable mate are high^4, 9^. Where pathogens can be transmitted through the resulting sexual contact, the same kinds of behavioural modulation can be adaptive for the pathogen^10^. This may be the case in the cricket *Gryllus texensis* where infection with a sexually-transmitted iridovirus counteracts illness-induced lethargy and causes earlier onset of courtship song^11^. In contrast, the effects of pathogen infection on same-sex sexual behaviour are unexplored, but such behaviours might also be modulated during infection, and any such modulations could affect the fitness of the infected individual or its pathogen in related ways.

The desert locust *Schistocerca gregaria*, and the entomopathogenic fungus *Metarhizium acridum*, provide a well-studied host-pathogen pair, because *M. acridum* is both a naturally occurring pathogen, and an important biological control agent^12^. Fungal conidia (asexual spores) deposited on the locust’s exterior germinate and then penetrate the cuticle, leading to systemic infection^12^. *Metarhizium* forms yeast-like blastospores, which disseminate the infection within the haemocoel and, in some *Metarhizium* species, secrete secondary metabolites that are toxic or interfere with the host immune response^12, 13^. If the host locust succumbs to infection and dies, mycelial growth of *Metarhizium* occurs and new conidia are produced on the surface of the cadaver^12^. During infection with *M. acridum*, locusts are known to undergo a number of behavioural changes caused by the infection itself, or in an attempt to combat it and enhance fitness. These include behavioural fever responses^14^, anorectic behaviour^15^, and reduced escape capability^16^.

Investigations using the locust-*Metarhizium* pathosystem often use only male locusts^13, 17, 18^, attempting to control for variability in size and mass between the sexes^17^. When housed in all-male groups, healthy male locusts are well known to mount other males^19, 20^. Among insects, such behaviours are often explained by imperfect sex recognition^2^. When this is the case, an animal’s mate acceptance threshold should be influenced by the costs of incorrectly accepting and incorrectly rejecting a mate^9^. To a locust in an all-male group, the probability of encountering a female would appear to be extremely low, so the costs of incorrectly accepting a mate are far less than the costs of incorrectly rejecting one. However, in the course of previous experiments on other aspects of immune-induced behavioural change (Clancy L., Ph.D. thesis, Aberystwyth University, 2015), we also noted that MMM appeared to be more common among *Metarhizium*-infected locusts than healthy controls. Therefore, we conducted experiments to verify this informal observation, and to investigate potential explanations for it.

Initially, we conducted quantitative observations of fungus-infected and control locusts over a four-week period, in order to verify whether MMM behaviour was more common among the infected individuals, and to provide data with which we could explore our hypothesis that such behaviours are a side-effect of terminal investment coupled with a lowered mate acceptance threshold. We then conducted experiments designed to verify trends from our first experiment in a different context, and to determine whether increased MMM behaviour in fungus-infected locusts might also be attributed to a reduction in the ability of locusts to fend off or escape MMM attempts due to infection.

## Results

**Figure 1:**
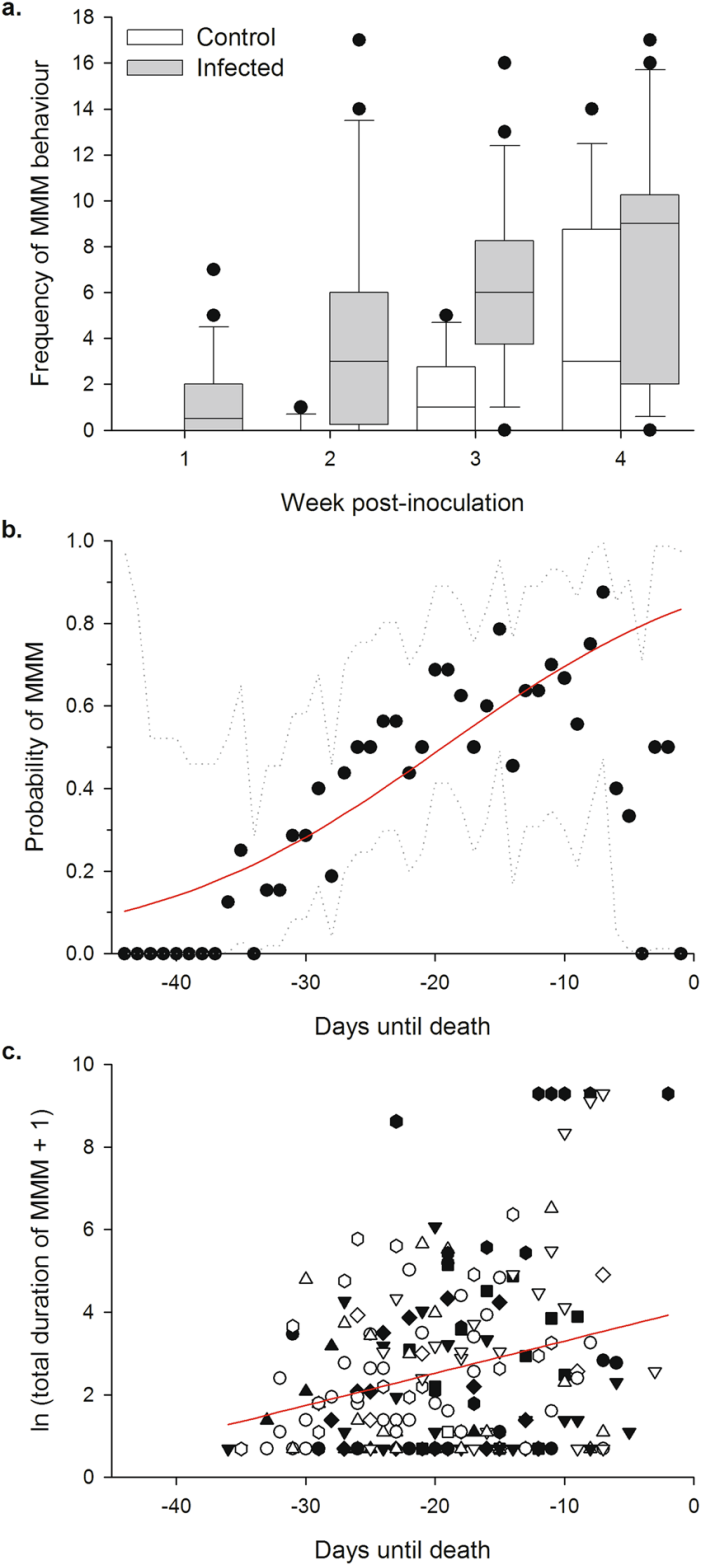
Male-male mounting behaviour in fungus-infected and control locusts. (**a**) Frequency of MMM behaviour observed in 24 fungus-infected and 12 control locusts over a 28-day period following inoculation. MMM behaviour frequencies were observed for 3 hours each day, and are summed over each week post-inoculation. Boxes denote the median, 25^th^ and 75^th^ percentile values, whiskers the 5^th^ and 95^th^ percentile values, and circles the more extreme observations. Note that two fungus-infected locusts died during the experiment, resulting in a sample size of 22 for that treatment in weeks three and four. (**b**) Probability of fungus-infected locusts mounting another male according to the time remaining until their death, for the 16 locusts from panel (**a**) that died during days 29–45 post inoculation. Points show the proportion of locusts performing at least one instance of MMM behaviour on a particular day before death. Because the sample size of locusts that were observed at a particular day before death varied according to their actual time of death, the resulting 95% binomial confidence intervals are plotted as dotted lines. The red line represents the fitted probability of performing MMM behaviour according to binary logistic GEE analysis (see text). (**c**) Natural log (x + 1) transformed duration of total time spent mounting other males for each locust that performed MMM behaviour on a particular day in panel (**b**), according to the time remaining until its death. Observations from the same individual have the same symbol and fill. Red line indicates the fitted relationship according to GEE analysis (see text).

We first made quantitative observations of desert locusts that were either *Metarhizium*-infected or control-treated and housed in all-male groups of the same treatment. Consistent with our informal observations, we found that instances of MMM increased over time for both treatment groups, but these behaviours occurred more frequently (and increased in frequency earlier in the experiment) in fungus-infected locusts (Negative binomial, log-link GEE using AR(1) working correlation matrix: Week: Wald X^2^_1_ = 164.441, p < 0.001; Infection status: Wald X^2^_1_ = 59.271; p < 0.001; Week*Infection status: Wald X^2^_1_ = 36.160, p < 0.001; see Fig. 1a).

We presumed that MMM events occurred in both groups due to the absence of females and a resulting decrease in mate acceptance threshold^9^. We hypothesised that the elevated frequency of MMM events in fungus-infected locusts might result from their proximity to death, causing a drive for terminal reproductive investment coupled with a further increase in the cost of incorrectly rejecting a female mate. In support of this, we found that the probability of a fungus-infected locust performing at least one mount of another male on a given day increased as the point of death approached (Binary logistic GEE using AR(1) working correlation matrix: Wald X^2^_1_ = 19.795; p < 0.001; see Fig. 1b). For those locusts that performed MMM behaviour, the total time spent in MMM events also significantly increased the closer they were to death (Gamma distribution, identity-link GEE using AR(1) working correlation matrix: Wald X^2^_1_ = 7.147; p = 0.008; see Fig. 1c).

**Figure 2:**
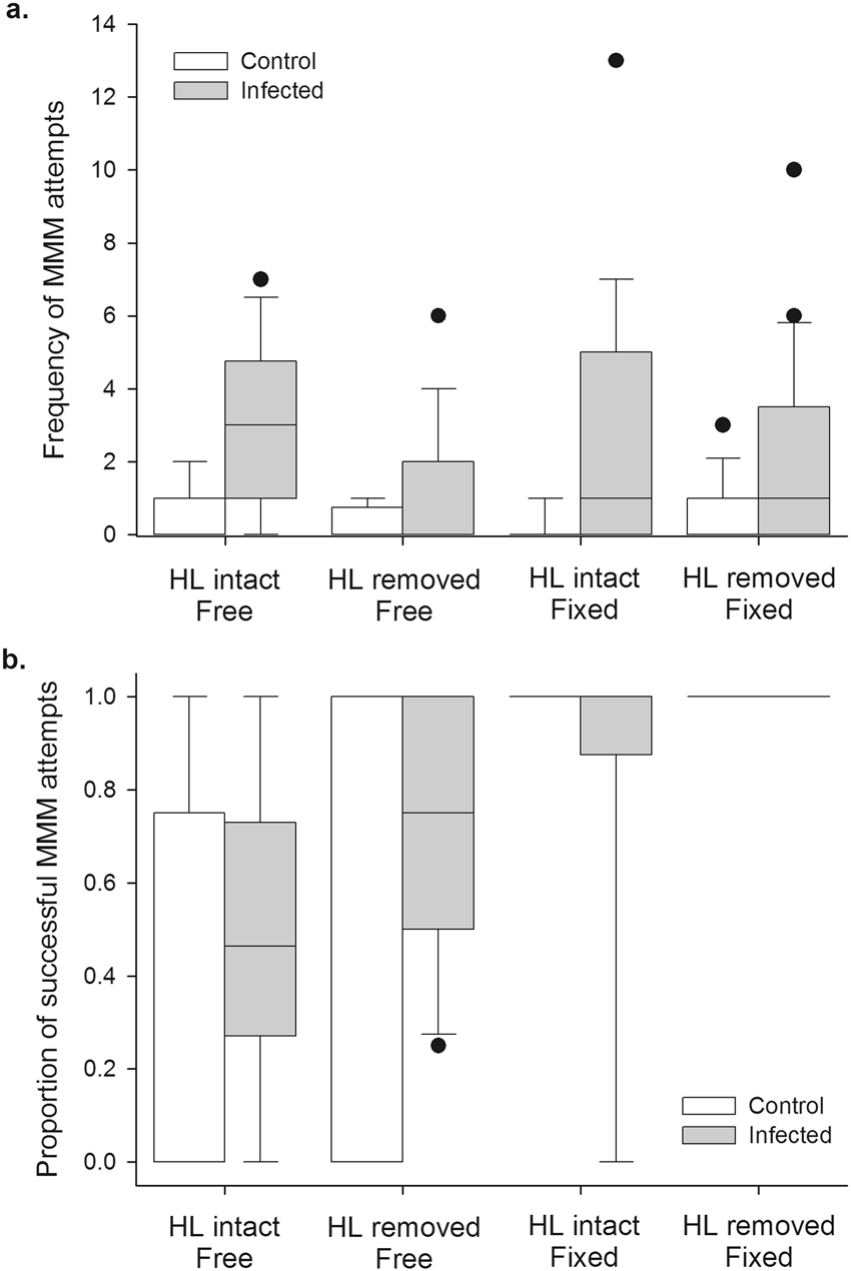
The effect of fungal infection on MMM attempts, and the effect of reduced escape capability on the success of MMM attempts. (**a**) Frequency of MMM attempts by control and fungus-infected focal locusts exposed to otherwise healthy males with hind legs removed, and/or fixed in position, such that they were less able to avoid or resist MMM attempts. Twenty-one control and 24 fungus-infected locusts were observed in total and exposed to each manipulated male type in random order. Due to locust deaths, data for a particular trial type could be missing for a given locust, and actual sample sizes were: HL intact/free – 19 control, 24 infected; HL removed/free – 20 cont., 21 inf.; HL intact/fixed – 19 cont., 23 inf.; HL removed/fixed – 18 cont., 21 inf. (**b**) Proportion of the MMM attempts in part (**a**) that resulted in the mounting male successfully positioning itself on top of the mounted locust in the manner characteristic of males mounting females. The sample comprised only those locusts that made at least one MMM attempt in part (**a**), and were: HL intact/free – 9 cont., 20 inf.; HL removed/free – 5 cont., 10 inf.; HL intact/fixed – 3 cont., 14 inf.; HL removed/fixed – 5 cont., 13 inf. For control locusts exposed to each type of free-moving manipulated male, the median values are zero; for infected locusts exposed to HL intact/fixed males,﻿ the median value is one. In both panels, boxes denote the median, 25^th^ and 75^th^ percentile values, whiskers the 5^th^ and 95^th^ percentile values, and circles the more extreme observations.

In order to confirm and further interrogate the patterns observed in our quantitative observations, we next experimentally manipulated the movement capability of healthy locusts, representing the reduced escape capability (‘lethargy’) that results from *Metarhizium* infection^16^. We then exposed fungus-infected and control-treated focal males to these manipulated males, and observed the interaction. During these trials, fungus-infected locusts attempted to mount the manipulated locusts significantly more frequently than did controls (Negative binomial distribution, log-link GEE using exchangeable working correlation matrix: Wald X^2^_1_ = 54.474; p < 0.001; see Fig. 2a). However, the frequency of these MMM attempts was not affected by whether or not the manipulated locust was restrained (Wald X^2^_1_ = 0.052; p = 0.820), or whether or not it had intact hind legs (Wald X^2^_1_ = 2.873; p = 0.090). Thus, a significantly higher frequency of MMM attempts was observed in fungus-infected locusts even when the mounted locusts were uninfected, verifying the pattern from our observational experiment and supporting our assertion that increased MMM attempts could be explained at least in part by fungal effects on the behaviour of the mounting locust.

From the same experiments we also investigated the probability that a MMM attempt by the focal locust would result in it successfully positioning itself on top of the manipulated locust (Fig. 2b). The infection status of the mounting locust was not a significant predictor of whether or not a MMM attempt would be successful (Binary logistic GEE using exchangeable working correlation matrix: Wald X^2^_1_ = 0.519; p = 0.471). However, if the manipulated locust was restrained, there was a significant increase in the proportion of attempted mounts that were successful (Wald X^2^_1_ = 32.797; p < 0.001; Fig. 2b). Whether or not the manipulated locust had intact hind legs (and therefore the ability to defensively kick) had no significant effect on MMM success (Wald X^2^_1_ = 2.613; p = 0.106; Fig. 2b). Thus, because fungal infection reduces mobility and escape capability^16^, this may also have contributed to the higher frequency and longer duration of MMM among infected locusts in our quantitative observations (Fig. 1).

## Discussion

In this study, we demonstrate that: (1) MMM is more common among fungus-infected than healthy male locusts, even when it is only the mounting locust that is infected; (2) the probability of MMM events occurring, and the amount of time spent in such behaviours, significantly increased with the mounting locust’s proximity to death; and (3) locusts with a reduced ability to escape were more likely to be successfully mounted. We speculate that the MMM behaviour observed here in female-deprived fungus-infected locusts may be due to increased terminal reproductive effort coupled with a lowered mate acceptance threshold in mounting males, and reduced escape capabilities in mounted males.

In our four-week observations, we observed MMM behaviours among infected and control locusts, and the instances of these behaviours increased over time for both groups. This finding is consistent with previous work on locusts housed in all-male groups^20^, and data from a wide range of taxa in biased sex ratio or single sex groups^21–24^. Such an effect is expected if an animal’s mate acceptance threshold is plastic, because the threshold should decrease when sex ratios are heavily biased and the costs of incorrectly rejecting a suitable mate are higher than those of incorrectly accepting an unsuitable one^9^. Consistent with this idea, male locusts emit a courtship-inhibiting pheromone that prevents rival males from displacing those guarding females, and may also help prevent same-sex sexual encounters. Males are more likely to ignore this pheromone the longer they experience female deprivation^25^. Thus, MMM behaviours in all-male locust groups are not unexpected.

In both of our experiments, we found an increase in MMM behaviour under fungal infection, and this has not been reported previously. This effect must result at least in part from fungal effects on the mounting locust, because fungus-infected locusts showed increased frequencies of MMM attempts on healthy locusts in our second experiment. In addition, we found significant increases in MMM occurrence and duration with the fungus-infected mounting locusts’ proximity to death in our first experiment. We suggest that two interacting factors may explain these patterns. First, theory suggests that individuals should increase their efforts to reproduce if their probability of being able to do so successfully in the future diminishes, leading to all-out terminal investment in reproduction for dying individuals^3, 5–8^. Alongside this effect, mate acceptance thresholds might be expected to lower, due to the increased costs of incorrectly rejecting a suitable mate, resulting in an increased likelihood of MMM behaviour^9^. In addition, male locusts may be more likely to ignore the courtship-inhibiting pheromones of other males due to their greater inclination to compete for mates^25, 26^.

In support of the concept of infection-induced terminal investment in reproduction, *M. acridum* infection has previously been shown to enhance maturation or egg laying in young adult locusts^27, 28^. In healthy female locusts, application of juvenile hormone (JH), or its analogues, enhances the rate of oocyte maturation and increases the number of developing eggs in the first maturation cycle^29^. Surgical removal of the corpus allatum in otherwise healthy males inhibits opposite-sex sexual behaviour and maturation-induced colour changes, but both sexual behaviour and colour changes are enhanced by subsequent JH applications^30^. For these reasons, it has been hypothesised that the adaptive increase in reproductive output that occurs during fungal infection may occur due to enhancement of JH synthesis^28^. Infection-induced enhancement of JH synthesis could also have caused the higher frequency of MMM events in our study. This could occur if locusts also had a low mate acceptance threshold due both to being housed in all-male groups, and being infected with a pathogen. In this way, increased MMM events could occur as a side effect of enhanced reproductive effort. In support of this suggestion, when male migratory locusts, *Locusta migratoria*, are deprived of females, treatment with a JH analogue has also been shown to enhance MMM behaviour in a manner resembling our results^20^.

Whilst misdirected terminal investment in reproduction might explain the actions of the mounting locust, it is less easy to understand the actions of the mounted individuals. During illness, many animals display characteristic sickness behaviours including lethargy, anorectic behaviour, and physiologically- or behaviourally-induced fever^31, 32^, although the expression of sickness behaviour can be modulated by social context (such as the availability of mates, for example)^33^. In our second experiment we found that experimentally manipulating a healthy male so that it could not move increased the probability that an attempt to mount it would be successful, whilst removing its ability to defensively kick did not. Under constant temperature conditions, *Metarhizium*-infected locusts move more than controls during the early stage of infection, presumably searching for warm microclimates to induce a behavioural fever^16^. However, during the final period of infection prior to death, infected locusts are less likely than controls to try to escape an approaching, startling stimulus, and move shorter distances when they do attempt to escape^16^. Although our experimental manipulations were more severe than would occur in all but the final stages of infection, our results suggest that the reduced defensive capabilities of fungus-infected locusts may contribute to the higher frequency and longer duration of MMM in that group.

Finally, enhancements in opposite-sex sexual behaviours in infected individuals appear to be adaptive for sexually-transmitted pathogens^10, 11^. MMM behaviour involving infected and uninfected individuals could not transmit *Metarhizium* in the same way since the infective conidia are not produced on the cuticle until after the death of the infected locust. However, such behaviour might cause infected individuals to act as vectors for ungerminated conidia on their body surface, similar to the exploitation of opposite-sex sexual behaviour in Diptera for the horizontal transmission of *Metarhizium anisopliae* conidia^34, 35^.

Taken together, our results demonstrate that fungus-infected male desert locusts housed in all-male groups are more likely to engage in MMM behaviour than controls under the same conditions. We suggest that this results from a combination of terminal reproductive drive in the mounting locust, coupled with a lowered mate acceptance threshold due to infection and the absence of females. However, we also suggest that reduced movement and defensive capability in infected locusts may increase their likelihood of being successfully mounted. Because our experiments investigated the unusual scenario of all-male groups, the occurrence of these trends in mixed-sex populations must now be examined in order to determine their significance for the fitness of infected locusts and their pathogens.

## Methods

### Locusts

Adult male desert locusts, *Schistocerca gregaria*, were purchased from Blades Biological Ltd (Edenbridge, Kent, UK) and housed in standard 330 × 200 × 410 mm aluminum locust cages. A 12:12 L:D regime was used with a controlled baseline temperature of 28 °C, and a thermal gradient of ~30–50 °C provided during the light period. Locusts were provided with water and wheat bran *ad libitum* and fresh wheat seedlings three times per week.

### Fungal culture and inoculation

*Metarhizium acridum* (ARSEF-324, obtained from the Agricultural Research Service Collection of Entomopathogenic Fungal Cultures (ARSEF), Ithaca, NY) was cultured on Potato Dextrose Agar; maintained at 28 °C; and kept under continuous light. Suspensions of fungal conidia in cottonseed oil were prepared to a concentration of 3.75 × 10^7^ per ml^13, 17^. Ten milliliters of cottonseed oil (Sigma-Aldrich, Dorset, UK) was added to 7-day old fungal cultures. Conidia were dislodged using a sterile spreader and the suspension was transferred to a sterile 25-ml bottle. The suspension was then filtered through four layers of sterile muslin to remove mycelia and clumps of conidia, centrifuged at 3000 rpm for 3 min, and then placed in a sonicating water bath at 15 °C for 5 min. The concentration of the conidial suspension was determined using a Neubauer haemocytometer and adjusted as required^13, 17^. Adult locusts were inoculated with 2 μl of fungal suspension (~75,000 conidia) under the pronotal shield using a micro-syringe (VICI®, Baton Rouge, Louisiana, USA). Controls were treated with 2 μl of cottonseed oil alone.

### Observation of MMM events

A total of 24 male locusts were inoculated with conidial suspension and 12 with cottonseed oil alone (the larger sample size of fungus-infected locusts facilitated an analysis of their behaviour with respect to their time of death; see below). Locusts were housed six of the same treatment to a cage. Each locust was uniquely identified with paint pens (POSCA, Mitsubishi Pencil Co. Ltd., Milton Keynes, UK), allowing us to track its behaviour over the course of the experiment. MMM behaviour was observed for 3 hours each day (11am to 2pm) from days 1–28 post-inoculation. Two fungus-infected locusts died during the experiment showing red colouration typical of *Metarhizium* infection, and subsequent sporulation. Only data from days 1–14 were analysed for those individuals. A mounting event was defined as a male locust positioning itself on the back of another male in the same orientation as occurs during male-female encounters. For each individual locust, the occurrence and duration of MMM behaviours were recorded.

Following the observation period, locust survival was monitored daily between day 29 and day 45 post-inoculation. During this period, 16 fungus-infected individuals died, allowing us to transpose the daily observations of their MMM behaviour to a scale of days until death. Fungus-infected locusts that did not die between days 29 and 45 post-inoculation could not be included in this analysis. Control locust MMM behaviour could not be analysed in this way because only two locusts died during the observation period.

### Experimental trials investigating MMM behaviour

To confirm trends from our four-week observations, and examine the impact of infection-induced reduction in defensive capability on the success of a mounting attempt, we conducted experimental trials using control-treated and fungus-infected locusts housed in all-male groups. We exposed 21 control and 24 fungus-infected focal locusts to each of four types of healthy males that had been manipulated to have a reduced capacity to escape or defend themselves. These male types were: (i) hind legs intact and free-moving; (ii) hind legs intact but fixed in position; (iii) hind legs removed but free-moving; and (iv) hind legs removed and fixed in position. Encounters were staged within semi-transparent plastic arenas (208 × 152 × 80 mm) with a 45-mm diameter mesh-covered hole in the lid to allow airflow but prevent escape. A curved wire climbing mesh (200 × 80 mm) was positioned in the arena, and manipulated males that were fixed in position were attached to this mesh using electrical wire. Experiments were conducted in a temperature-controlled growth room at 28 °C. Focal locusts were exposed to each manipulated male type in random order between days 10 and 17 post-inoculation, though occasionally individual trials could not be conducted due to locust deaths. Each trial lasted 7 hours, during which, attempts to mount the manipulated male and successful MMM behaviours (as defined above) were recorded.

### Statistical methods

Generalised Estimating Equations (GEEs) were employed to investigate repeated observations from the same individuals^36^. For experiments where repeated observations were ordered in time, the AR(1) working correlation matrix was used; for manipulated male trials where treatments were randomized to control for patterns in time, an exchangeable working correlation matrix was used.

We employed negative binomial distribution, log-link function GEEs to analyse counts of discrete MMM events from our initial experiment, using infection status as a factor and experimental week as a covariate. In our reanalysis of these data according to each locust’s proximity to death, we employed a binary logistic GEE to analyse whether or not a locust performed MMM behaviour on a given day (with the response coded as either no mounting event occurring on a particular day [0], or one or more mounting events occurring on a particular day [1]). We analysed the total duration of time per day that those locusts who performed MMM behaviour spent doing so using a gamma distribution, identity link GEE model using ln(x + 1) transformed duration data. This was done because the raw data were not normally distributed, but transformation could not sufficiently restore normality.

In manipulated male experiments, counts of MMM attempts were analysed using a negative-binomial, log-link GEE, and whether or not these attempts were successful was analysed using a binary logistic GEE model. In both cases, infection status, whether or not the manipulated male had intact hind legs, and whether or not the manipulated male was mobile, were entered as factors in the analyses.

All statistical analyses were conducted using SPSS version 2.0.0.2 (IBM Corp., Armonk, NY, USA). The binomial confidence intervals plotted in Fig. 1 were calculated using the confint.xla add-in for Microsoft Excel^37^.

### Data Availability

All data generated and analysed during this study are provided in Supplementary Table S1.

## Electronic supplementary material

Table S1
